# Human γδ T-Cells: From Surface Receptors to the Therapy of High-Risk Leukemias

**DOI:** 10.3389/fimmu.2018.00984

**Published:** 2018-05-07

**Authors:** Vito Pistoia, Nicola Tumino, Paola Vacca, Irene Veneziani, Alessandro Moretta, Franco Locatelli, Lorenzo Moretta

**Affiliations:** ^1^Immunology Area, IRCCS Bambino Gesù Pediatric Hospital, Rome, Italy; ^2^Dipartimento di Medicina Sperimentale and Centro di Eccellenza per le Ricerche Biomediche, Università degli Studi di Genova, Genoa, Italy; ^3^Department of Onco-Hematology and Cell and Gene Therapy, IRCCS Bambino Gesù Pediatric Hospital, Rome, Italy; ^4^Department of Pediatric Science, University of Pavia, Pavia, Italy

**Keywords:** γδ T-cells, receptors, hematopoietic stem cells, HLA-haploidentical transplantation, αβ T-cell, B-cell depletion

## Abstract

γδ T lymphocytes are potent effector cells, capable of efficiently killing tumor and leukemia cells. Their activation is mediated by γδ T-cell receptor (TCR) and by activating receptors shared with NK cells (e.g., NKG2D and DNAM-1). γδ T-cell triggering occurs upon interaction with specific ligands, including phosphoantigens (for Vγ9Vδ2 TCR), MICA-B and UL16 binding protein (for NKG2D), and PVR and Nectin-2 (for DNAM-1). They also respond to cytokines undergoing proliferation and release of cytokines/chemokines. Although at the genomic level γδ T-cells have the potential of an extraordinary TCR diversification, in tissues they display a restricted repertoire. Recent studies have identified various γδ TCR rearrangements following either hematopoietic stem cell transplantation (HSCT) or cytomegalovirus infection, accounting for their “adaptive” potential. In humans, peripheral blood γδ T-cells are primarily composed of Vγ9Vδ2 chains, while a minor proportion express Vδ1. They do not recognize antigens in the context of MHC molecules, thus bypassing tumor escape based on MHC class I downregulation. In view of their potent antileukemia activity and absence of any relevant graft-versus-host disease-inducing effect, γδ T-cells may play an important role in the successful clinical outcome of patients undergoing HLA-haploidentical HSCT depleted of TCR αβ T/CD19^+^ B lymphocytes to cure high-risk acute leukemias. In this setting, high numbers of both γδ T-cells (Vδ1 and Vδ2) and NK cells are infused together with CD34^+^ HSC and may contribute to rapid control of infections and leukemia relapse. Notably, zoledronic acid potentiates the cytolytic activity of γδ T-cells *in vitro* and its infusion in patients strongly promotes γδ T-cell differentiation and cytolytic activity; thus, treatment with this agent may contribute to further improve the patient clinical outcome after HLA-haploidentical HSCT depleted of TCR αβ T/CD19^+^ B lymphocytes.

## General Features of Human γδ T-Cells

γδ T-cells straddle the innate and adaptive arms of the immune system and are involved in response to pathogens [e.g., mycobacteria; cytomegalovirus (CMV)] and tumors. Similar to αβ T-cells, γδ T-cells develop in the thymus, but express a rearranged T-cell receptor (TCR) consisting of a TCR-γ and a TCR-δ chain (1). Although important information has been obtained by studies in mice, this review will be focalized on human γδ T-cells (2). Four human γδ T-cell populations can be identified by the TCR Vδ expression (Vδ1, Vδ2, Vδ3, and Vδ5) (3). Vδ1, Vδ2, Vδ3, and Vγ2, Vγ3, Vγ4, Vγ5, Vγ8, Vγ9, and Vγ11 are the most frequently gene segments used in rearrangement of δ and γ chains, respectively (4). In humans, most peripheral blood γδ T-cells express Vδ2 TCR chain paired with Vγ9 chain (5), while γδ T-cells expressing Vδ1 or Vδ3 TCR chain can be paired with various Vγ chains (6) and they are predominant in epithelial tissues of skin, lungs, intestine and reproductive tract (7), liver, spleen, and thymus (8). γδ T-cells with Vγ4Vδ5 TCR are able to bind the endothelial protein C receptor (9). Moreover, four subsets of γδ T-cells were detected (Vδ4, Vδ6, Vδ7, and Vδ8) in peripheral blood of patients with B-cell non-Hodgkin lymphoma, but the γ chain pairings are still unknown. Although the majority of γδ T-cells do not express either CD4 or CD8, there is a small percentage of γδ T-cells that are CD8 positive (8). Different mechanisms of TCR rearrangement occur in mouse immune system (2).

The major pathways of γδ T-cell activation involve triggering of the γδ TCR that, at variance with αβ T-cells, does not recognize peptides presented by antigen-presenting cells (APCs) in the context of the MHC. The γδ TCR may bind soluble or membrane proteins, such as tetanus toxoid (10), bacterial proteins (11), viral proteins (2), and heat shock proteins. Moreover, the γδ TCR may bind CD1d expressed by professional antigen presenting cells (APCs), presenting glycolipids and microbial lipids (12).

In adult human, γδ T-cell population represents 1–5% of all CD3^+^ cells. In peripheral blood of healthy human subjects, T-cells expressing Vγ9Vδ2 TCR can account for up 95% of γδ T-cells (10) and render between 1 and 10% of all blood T-cells (2). Conversely, Vδ1 T-cells represents only 10–30% of γδ T-cells in peripheral blood of healthy human (10). In the lymphoid tissue and in the gut- and skin- associated lymphoid systems, γδ T-cells show a frequency similar to that detected in peripheral blood (11). Vγ9Vδ2 T-cells are activated (13, 14) by natural metabolites known as phosphoantigens (PhAgs), such as isopentenyl pyrophosphate (IPP), produced in eukaryotes through the mevalonate pathway involved in cholesterol synthesis and protein prenylation (15). A dysregulated mevalonate pathway leading to overproduction of endogenous IPP occurs in transformed cells (16, 17). The endogenous production of IPP and related pyrophosphates and the consequent ability of a given cell type to activate γδ T-cells can be pharmacologically manipulated. A critical enzyme in the mevalonate pathway is farnesyl pyrophosphate synthase (FPPS), which acts downstream of IPP production. Targeted knockdown of FPPS leads to accumulation of IPP and subsequent activation of γδ T-cells (18). Treatment of tumor cells or monocytes with the bisphosphonate zoledronic acid (ZOL), which blocks FPPS function, leads to increased IPP production, and thereby induces selective activation of Vγ9Vδ2 T-cells (17, 19, 20). Until recently, it was unclear how the Vγ9Vδ2 TCR could recognize PhAgs. This enigma has been clarified by the discovery that butyrophilin 3A1 (also known as CD277) plays an essential role in the interaction of PhAgs with the Vγ9Vδ2 TCR, although the fine mechanisms of the phenomenon are still to be fully elucidated (4, 21).

Upon activation, γδ T-cells can produce large amounts of Th1 cytokines, such as IFNγ and TNFα, and directly induce monocyte-derived dendritic cell maturation and activation, suggesting a potential adjuvant role of this cross-talk in enhancing antigen-specific αβ T-cell response (12, 13). In this respect, it has been reported that γδ T-cells may take up and process soluble proteins inducing proliferation, cytokine production and cytotoxicity by CD8^+^ αβ^+^ T-cells (22).

The ability of γδ T-cells to kill hematological and solid tumors and to release Th1-type cytokines, combined with the possibility of growing these cells in culture, has attracted great interest for their use as adoptive cell therapy of cancer. Emphasis has been placed on Vγ9Vδ2 T-cells, which are easily expanded *in vitro* by PhAg stimulation (induced by exposure of cells to ZOL) and can be further boosted *in vivo* with ZOL or other synthetic PhAgs. Several clinical trials of Vγ9Vδ2 T-cell-based immunotherapy for both hematological malignancies (23–26) and solid tumors (27–32) have been conducted with promising results. A note of caution on the efficacy of these approaches comes from the plasticity of γδ T-cells controlled by the signals from the microenvironment, which can switch the antitumor profile of these cells to a tumor-promoting one, for example through induction of IL-17 production (33).

## γδ T-Cells: Receptors and Ligands

**Figure 1:**
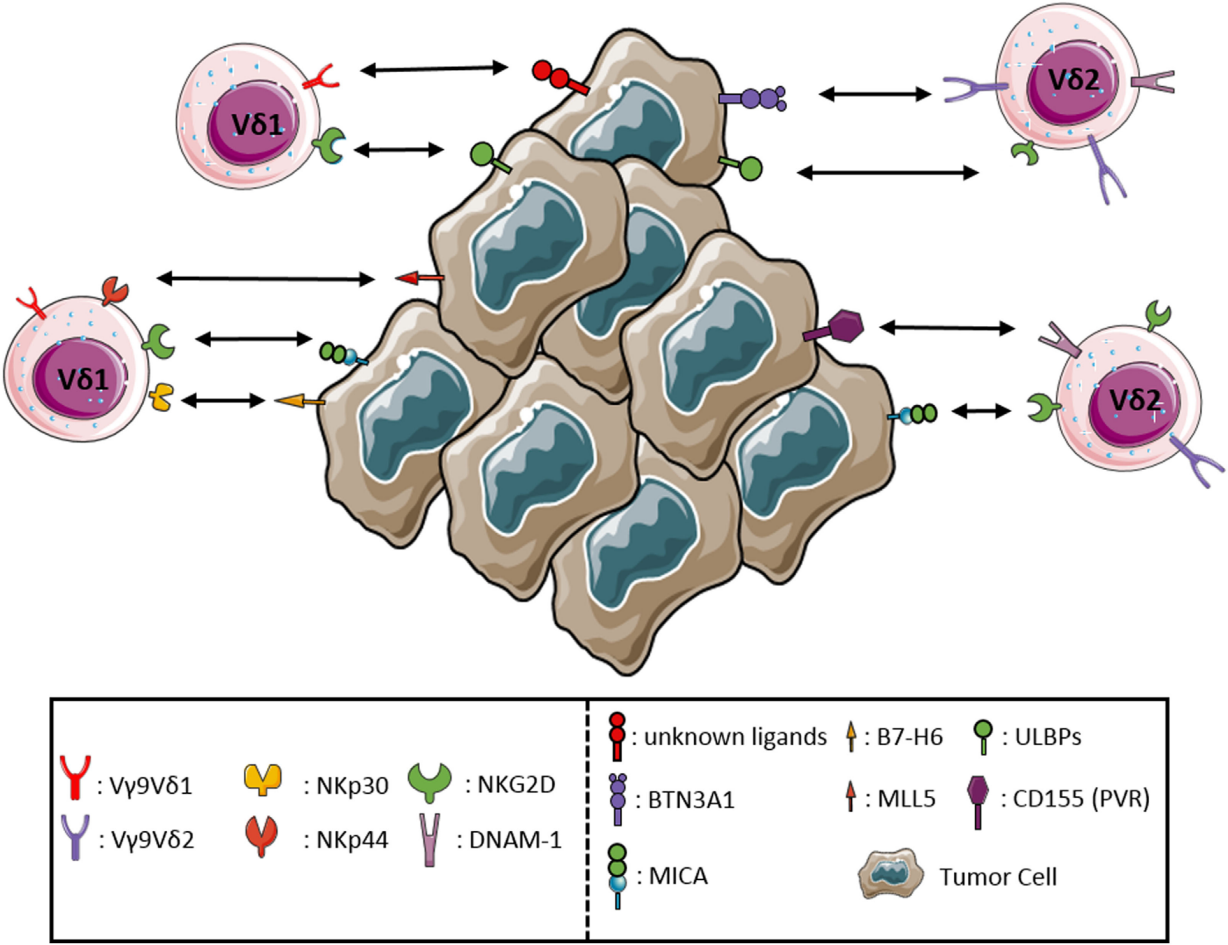
Receptor–ligand interactions between γδ T lymphocytes and tumor cells. The major interactions occurring between the activating receptors expressed by γδ T lymphocytes and the corresponding ligands either *de novo* expressed or upregulated by tumor cells are represented in detail.

A feature typical of NK cells shared by γδ T-cells is the ability to kill malignant and infected cells in the absence of any prior exposure. Moreover, γδ T-cells share with NK cells the expression of different NK receptors (NKRs), such as the NK activating receptor DNAM-1, the Fc receptor CD16, and the C-type lectin-like receptor NKG2D (34). Tumor cell recognition and the associated γδ T-cells activation require the engagement of the TCR and/or NKRs, mostly NKG2D. NKG2D binds MHC class I polypeptide-related sequence MICA, MICB, and UL16 binding proteins (ULBPs) expressed on stressed and tumor cells. Overexpression of the NKG2D ligands ULBP1 and ULBP4 (35) by hematological and epithelial tumors, respectively, drives efficient cytotoxic responses by Vγ9Vδ2 T-cells. The proteins that can induce Vδ1 activation are incompletely known, although CD1c and CD1d, members of CD1 family, can activate Vδ1 T-cells through TCR binding (36). Vδ1 T-cells of the human intestinal epithelium are able to recognize MICA and MICB ligands, by the synergistic actions of TCR and NKG2D. Moreover, in Vδ1 T-cells subset, the interaction of NKp30 with B7-H6, expressed on tumor cells, allows a specific antitumor activity (9). Both TCR and NKG2D bound overlapping fragments of MICA, with different affinity and kinetics, the affinity of NKG2D being by far superior to that of TCR (37). The TCR–MICA complex was particularly stable, suggesting a sequential model, whereby the initial binding of NKG2D is followed by the formation of the more stable TCR–MICA complex. MICA engagement by TCR was found to be indispensable for γδ T-cell-mediated cytotoxicity, while NKG2D played a co-stimulatory role (38). ULBP molecules may be recognized in a similar manner, as it has been shown that ULBP4 engages both NKG2D, and Vγ9Vδ2 TCR. DNAM-1, another NKR involved in activation of Vγ9Vδ2 T-cells, binds its ligand nectin-like 5 on tumor cells rapidly triggering the cytotoxic activity of Vγ9Vδ2 T-cells (39). Controversial results have been reported regarding the expression and function of NKp44 on a minor subset (less than 10%) of γδ T-cells after culture in the presence of IL-15 (40). In addition, some γδ T-cells may express the HLA-E-specific CD94/NKG2A inhibitory receptor. Thus, following interaction with HLA-E^+^ cells, the functional activity of these cells may be modulated, as reported in the case of γδ T-cells interacting with enterocytes (41). The sequential recognition of different targets by γδ T-cells could play an important role in immunosurveillance, as it allows the latter cells to rapidly scan target cells for stress markers indicative of possible infection or malignant transformation. The requirement for a multicomponent stress context for full γδ T-cell activation could then provide fail-safe protection against autoimmunity. The apparent co-existence of diverse co-stimulatory axes decreases the chances of immune evasion. The main interactions between γδ T-cells and tumor cells are shown in Figure 1.

## γδ T-Cells and Hematopoietic Stem Cell Transplantation (HSCT)

**Figure 2:**
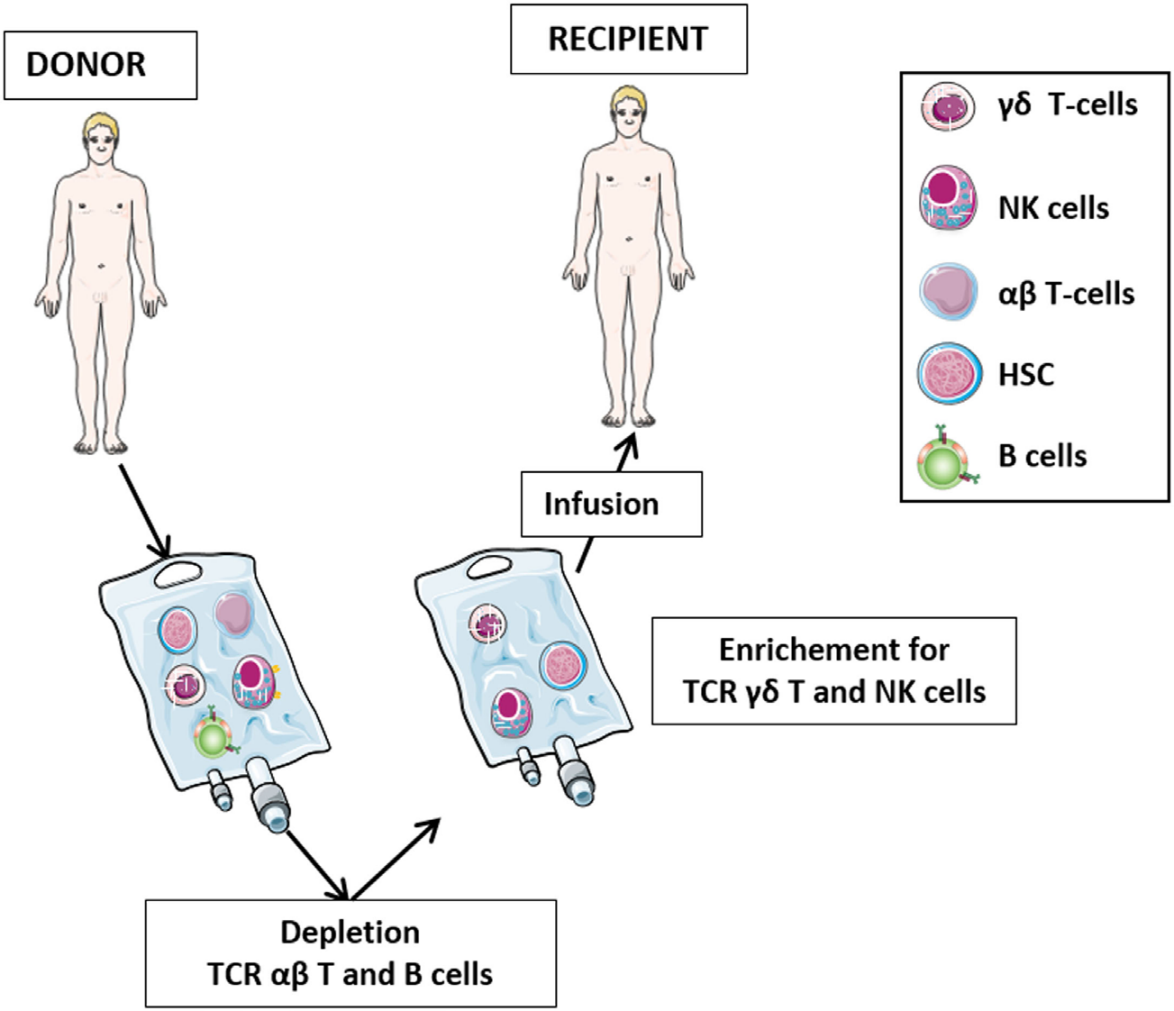
The protocol of T-cell receptor (TCR) αβ T-cell and B-cell depletion recently developed in haplo-HSC transplantation. The protocol for haplo-HSCT allows infusion of donor HSC, together with high number of γδ T lymphocytes and NK cells. This novel strategy permits, during the early posttransplant period (6–8 weeks), a better control of leukemia cells escaping the preparative regimen, thus preventing disease relapses. In this transplantation setting, in addition to γδ T-cells and NK cells developing late from donor HSC, these promptly available effectors transferred with the graft may immediately exert their graft-versus-leukemia effect and contribute to the control of infections.

The role of γδ T-cells in HSCT has been the subject of numerous studies in the last three decades (Figure 2). After initial reports with contrasting results (42–44), it was demonstrated that 5-year disease-free and overall survival of leukemia patients who received HLA-mismatched allo-HSCT depleted of TCR αβ T-cells correlated significantly with high number of γδ T-cells circulating in patient peripheral blood after transplantation (45–47). It was proposed that γδ T-cells, recovering after the allograft, play a relevant role in the graft-versus-leukemia (GvL) (46), albeit other studies have highlighted the prominent GvL activity of NK cells in T-cell-depleted HSCT (48, 49). Analysis of the TCR Vδ repertoire revealed that circulating Vδ1 cells are predominant in patients with high γδ T-cells counts, whereas patients with low γδ T-cells counts and healthy individuals display mostly Vδ2 cells (46).

A study carried out at a single institution investigated the clinical impact of γδ T-cell reconstitution in 102 consecutive pediatric patients with acute leukemia given allogeneic HSCT from different donors and employing different cell sources (50). A major finding was that the probability of infections in patients with high counts of circulating γδ T-cells after the allograft was significantly lower than that in patients with low or normal counts of γδ T-cells. In particular, no bacterial infection occurred in the former patient group. Furthermore, also event-free survival of patients with high numbers of circulating γδ T-cells after HSCT was significantly better than that of patients with low or normal numbers of γδ T-cells (50).

It is still a matter of debate whether γδ T-cell regeneration in HSCT recipients occurs either *via* the peripheral expansion of mature donor T-cells present in the graft or through a differentiation process from donor HSC. Using polymerase chain reaction-based complementarity-determining region (CDR)3 spectratyping and DNA sequencing for TCR δ chains, it was initially shown that the size distribution patterns of CDR3 were recovered a few months after allo-HSCT and that such recovery was faster than that of αβ T-cells (51). Clonal predominance of TCR Vδ1^+^ cells occurred after transplantation in a few patients, and follow-up of a donor-recipient pair supported the hypothesis that peripheral expansion of mature donor T-cells contained in the graft was the main pathway of γδ T-cell regeneration after allo-HSCT (51). More recently, a study has evaluated human γδ T-cell reconstitution using an RNA and cDNA-based next generation sequencing (NGS) approach that has allowed the investigation at the clonal level of TCR γ and δ chain (TRG and TRD) repertoires in sorted γδ T-cells before and after allo-HSCT (52). In the absence of CMV reactivation, such repertoires developed after 30–60 days from allo-HSCT and remained stable over at least 6 months. TRG and TRD repertoires after transplantation were qualitatively comparable to those present before transplantation, but contained clonotypes different from those found in the donor, suggesting that they were generated *de novo* from donor HSC through a process of cell maturation. In addition, reactivation of CMV caused massive perturbations of TRG and TRD repertoires, being associated with preferential proliferation and expansion of a few individual Vδ1 and Vδ3 T-cell clones. Taken together, these studies indicate that γδ T-cells are capable of adaptive responses generating different TRG and TRD repertoires and different clonal expansions (51, 52).

## γδ T-Cells and αβ T-Cell-Depleted HLA-Haploidentical HSCT

Hematopoietic stem cell transplantation from an HLA-haploidentical relative (haplo-HSCT) provides most patients lacking a suitable matched donor with the chance of undergoing transplantation. Clinical development of haplo-HSCT has been boosted by the demonstration that extensive T-cell depletion from the graft efficiently prevents both acute and chronic graft-versus-host disease (GvHD), even when the donor and the recipient were mismatched for an entire HLA haplotype (53, 54). The therapeutic efficacy of T-cell-depleted haplo-HSCT largely depends on donor NK cells either generated from HSC or infused with the graft mediating a potent GvL effect (55–58). Such effect is delayed in patients transplanted with positively selected donor CD34^+^ cells, since the first wave of killer-immunoglobulin-like receptor-positive, alloreactive NK cells, differentiating from infused HSC appears after a minimum time interval of 6–8 weeks (49, 56, 59, 60). The delayed availability of mature NK cells, mainly responsible of GvL effect, explains, at least in part, the transplant-related mortality and the early leukemic relapses, particularly in acute myeloid leukemia (AML). To circumvent this problem, a novel strategy of graft manipulation has been set up, whereby both T-cells bearing the αβ T-cell receptor (TCR) and CD19^+^ B lymphocytes are depleted from the graft before infusion (61, 62). This approach abates TCR αβ T-cell-mediated GvHD, prevents Epstein–Barr virus-driven B cell lymphoproliferative disorders occurring in immunocompromised patients, and allows to transfer to the recipient high numbers of haploidentical CD34^+^ cells and of mature NK cells and TCR γδ T-cells, which can readily exert protective functions against leukemia cell re-growth and life-threatening infections (63–66). Notably, TCR γδ T-cells and NK cells share a number of features that are relevant in the haplo-HSCT setting. Both cell types: (i) kill tumor cells in a MHC-independent manner (34), (ii) are involved in anti-CMV immune responses (67), (iii) do not mediate GvHD, since they do not recognize peptide antigens presented in the context of MHC (12), (iv) interact with each other and with additional immune cells, such as αβ T-cells and dendritic cells (12), and (v) following activation, are cytotoxic to mesenchymal stromal cells, a major component of tumor microenviroment (68).

We have recently investigated γδ T-cell reconstitution after haplo-HSCT depleted of TCR αβ^+^ T/CD19^+^ B cells (Figure 2) in 27 children, 15 of whom had leukemia and 12 primary immune-deficiencies or bone marrow failure syndromes (63). Immunophenotypic characterization of peripheral blood mononuclear cells performed at 1, 3, and 6 months after transplantation showed an initial predominance of γδ over αβ T-cells, followed by progressive recovery of the latter cells. γδ T-cells included three different populations, i.e., Vδ2, Vδ1 and, at a lower extent, Vδ2^−^, Vδ1^−^ (63). Four subsets of human γδ T-cells have been identified based upon the expression of the CD45 and CD27 surface markers: *naïve* (CD45RA^+^, CD27^+^), central memory (CM: CD45RA^−^, CD27^+^), effector memory (EM: CD45RA^−^, CD27^−^), and terminally differentiated (EMRA: CD45RA^+^, CD27^−^) (69, 70). Similar to the corresponding αβ T-cell subsets, *naïve* and CM γδ T-cells express lymph-node homing receptors and are devoid of immediate effector functions. In contrast, EM and EMRA γδ T-cells express receptors for migration to inflamed tissue where they mediate effector functions, such as cytotoxicity and cytokine production (70). Studies carried out on Vγ9Vδ2 T-cells have demonstrated that PhAg-stimulated *naïve* cells generate TCM cells, while cytokine-stimulated TCM cells differentiate into TEM or TEMRA in the absence of antigen (69). Notably, TEMRA Vγ9Vδ2 T-cells are the major subset endowed with potent antitumor and antibacterial activity (69). Analysis of the differentiation status of γδ T-cells in our patients given haplo-HSCT showed that TCM cells were predominant in both Vδ2 and Vδ1 cells. The relative proportions of the different Vδ2 and Vδ1 subsets remained stable over time and were similar to those detected in the donor. *Naïve* Vδ2 cells increased significantly between 20 days and 3 months after haplo-HSCT, suggesting that circulating γδ T-cells in transplanted patients consisted of not only mature cells derived from the graft, but also of cells differentiating from donor HSC (63). Investigation of TRG and TRD repertoires in recipients of haplo-HSCT depleted of TCRαβ^+^ T and CD19^+^ cells using powerful NGS techniques will shed new light on the origin of γδ T-cells in this setting.

Studies performed in solid organ transplantation and HSCT recipients have demonstrated that a remarkable expansion of Vδ2^−^ γδ T-cells displaying a TEMRA immunophenotype and exerting cytotoxic function takes place in the course of CMV infection (67). The investigation of the γδ TCR junctional diversity revealed the expansion of Vδ1 and Vδ3 T-cells with a restricted repertoire during CMV infection (67). The mechanism whereby Vδ2- γδ T-cells recognize CMV-infected cells involves γδ TCR, still incompletely defined co-stimulatory molecules including LFA-1, and different γδ TCR ligands expressed by virus-infected cells (67). One of such ligands is the recently identified MHC-related molecule endothelial protein C receptor (71). CMV-induced Vδ2 γδ T-cells are able to recognize and kill hematological tumor cell lines and primary AML blasts (72, 73). Consistently with these notions, our patients who experienced CMV reactivation displayed a significant expansion of the Vδ1 T-cell subset with a cytotoxic TEMRA phenotype, which was absent in patients without CMV reactivation. These CMV-driven Vδ1 T-cells killed *in vitro* primary acute lymphoblastic leukemia and AML blasts more efficiently than Vδ1 T-cells from patients that did not reactivate CMV infection, suggesting that CMV infection promotes both expansion and activation of Vδ1 T-cells (63).

## Effect of Zoledronic Acid on γδ T-Cells Recovering after αβ T-Cell-Depleted HLA-Haploidentical HSCT

We demonstrated that Vδ2 T-cells from patients who received haplo-HSCT depleted of TCRαβ^+^ and CD19^+^ cells expanded *in vitro* upon incubation with ZOL, which promoted the acquisition of an EM phenotype and potentiated the cytotoxic activity against primary leukemic blasts. Such activity was dependent on the levels of PhAgs expressed by leukemia cells and on TCR Vγ9 mediated recognition of the latter cells (63). Indeed, the lytic capacity of γδ T-cells was strongly enhanced by sensitizing leukemic target cells with ZOL. These *in vitro* results provided the rationale to investigate in a subsequent clinical study the effect of ZOL infusion in 43 pediatric recipients of haplo-HSCT depleted of TCRαβ^+^ and CD19^+^ cells (74). ZOL was infused every 28 days at least twice in most patients. Such treatment was safe and well tolerated, and, when administered three or more times, reduced GvHD occurrence and improved overall survival. The first treatment with ZOL induced the differentiation of Vδ2 T-cells, which switched from a CM to an EM/EMRA phenotype. Such maturation correlated with increased Vδ2 cell-mediated cytotoxicity against primary leukemia cells irrespective of their PhAg expression. Proteomic analyses identified an anti-proliferative effect of infused ZOL on total γδ T-cells that was consistent with the decrease of Vδ2 T-cells starting 3 months after HSCT. Such effect was already evident after the first ZOL infusion and it was further boosted by the subsequent infusions. In contrast, the percentage of Vδ1 T-cells increased during ZOL infusions irrespective of CMV reactivation (74). Altogether, these results suggest that haplo-HSCT transplanted pediatric patients may benefit from ZOL treatment.

## Conclusion

Similar to NK cells, γδ T-cells are endowed with antileukemia and anti-infection potential and do not mediate GvHD. These features are particularly useful in the setting of haplo-HSCT depleted of TCR αβ^+^ T and CD19^+^ B cells, since the graft infused into the patient is highly enriched in mature γδ and NK cells ready to exert their effector functions. Both Vδ2 and Vδ1 γδ T-cells are cytotoxic toward primary acute leukemia cells, while Vδ1 and Vδ3 cells undergo adaptive clonal expansions driven by CMV reactivation that are reminiscent of antigen-specific αβ T-cells responses. Pharmacological manipulation, for example, through ZOL administration, may potentiate the anti-leukemic activity of endogenous Vδ2Vγ9 T-cells; if this effect translates into a significant benefit for the patients awaits to be definitively proved in prospective controlled clinical trials. Future studies aimed at deconvoluting the fine mechanisms whereby γδ T-cells recognize malignant and virus-infected cells will help improve the therapeutic potential of γδ T-cells in the setting of haplo-HSCT.

## Author Contributions

All authors discussed together the general outline of the article. VP, NT, and LM wrote the first draft that was subsequently reviewed by PV, IV, AM, and FL. Thereafter, all authors contributed to the elaboration of the final version of the manuscript.

## Conflict of Interest Statement

The authors declare that the research was conducted in the absence of any commercial or financial relationships that could be construed as a potential conflict of interest. The reviewer EP and handling Editor declared their shared affiliation.
